# Home-Based Dynamics of Sleepiness-Related Conditions Starting at Biological Evening and Later (Beyond Working)

**DOI:** 10.3390/ijerph20176641

**Published:** 2023-08-24

**Authors:** Valeriia Demareva, Irina Zayceva, Valeriia Viakhireva, Marina Zhukova, Ekaterina Selezneva, Ekaterina Tikhomirova

**Affiliations:** Faculty of Social Sciences, Lobachevsky State University of Nizhny Novgorod, 603022 Nizhny Novgorod, Russia

**Keywords:** sleepiness, ‘drowsy’ condition, ‘stressed’ condition, biological evening, circadian system, shift work

## Abstract

Shift work requires round-the-clock readiness to perform professional duties, and the workers’ performance highly depends on their sleepiness level, which can be underestimated during a shift. Various factors, including the time of day, can influence sleepiness in shift workers. The objective of this study was to explore the dynamics of sleepiness-related conditions assessed through heart rate variability analysis, starting from the biological evening and continuing in vivo (at home), without the need for artificial alertness support. The participants solely performed regular evening household duties. A total of 32 recordings were collected from the Subjective Sleepiness Dynamics Dataset for analysis. At 8:00 p.m. and every 30 min thereafter, the participants completed cyclic sleepiness scales (the KSS and the SSS) until the time they went to bed, while their heart rate was recorded. The results of the study indicated that during the biological evening, high sleepiness is associated with a ‘stressed’ condition characterized by higher sympathetic activation. Later on, it is associated with a ‘drowsy’ condition characterized by higher parasympathetic activation and a decline in heart rate variability. Our findings provide evidence that the type of condition experienced during high sleepiness depends on the biological time. This should be taken into account when managing work regimes in shift work and developing alertness detectors.

## 1. Introduction

There are numerous professions that require round-the-clock readiness to perform duties. They are usually organized as shift work. In most cases, shift work involves professional activities not only during the day but also in the evening and at night. It is worth noting that shift work is often used in emergency response services and in complex industries where a high level of worker vigilance is important to eliminate errors due to human factors. Research shows that there are differences in a person’s conditions during day and night shifts [1,2,3].

These differences are associated with the natural dynamics of sleepiness levels throughout the day: regardless of sleep time, sleepiness gradually decreases in the morning and gradually increases towards the evening, reaching its peak at night [4,5]. Sleepiness is defined as a natural biological function determined by the likelihood of falling asleep [6] and the tendency to doze off or fall asleep when a person intends to be awake [7]. Subjective sleepiness is usually measured using the Stanford Sleepiness Scale (SSS) [8] and the Karolinska Sleepiness Scale (KSS) [9].

Increased sleepiness is associated with the occurrence of critical errors in activity, including those leading to accidents, such as driving a car [10]. During the study of a person’s conditions during night shifts, a decrease in psychomotor vigilance [2] and sensitivity to visual search [11] has been identified. This suggests that peaks and dips in subjective sleepiness ratings coincide with objective indicators of impaired performance [12]. Night shifts have been shown to result in decreased productivity, manifested by increased reaction time [1] and an increase in serious errors [3]. Similar trends persist across different professions: helicopter pilots [13], fishermen [3], healthcare workers [2,11], and metallurgists [1]. Subjective vigilance and neurobehavioral indicators are also impaired in simulated night shift conditions [14]. Subjective sleepiness is associated with a loss of productivity: subjective sleepiness levels increased 2–3 h before a loss of performance and 4–6 h before physiological sleepiness signs [15].

Various factors can influence sleepiness in shift workers. In a night schedule, there is a tendency for maximum levels of subjective sleepiness and low subjective vigilance on the first night of work [1,11,13]. However, other studies show that on subsequent nights, participants were unable to detect a decrease in vigilance, although objective factors indicated its loss [2]. The environment in which people are situated also affects their condition. For example, sleepiness and physical fatigue levels in fishermen were dependent on the nature of the vessel’s movement [3], and regulating the level of illumination helped shift the timing of the steady urge to sleep [14]. Age is also a significant factor: in drowsy conditions, elderly drivers did not increase the number of errors as much as young drivers, who were 11 times more likely to be involved in dangerous situations leading to accidents compared to when they were alert [10]. Another important factor is the time of day. The evening serves as a natural transitional time between low and high sleepiness and, therefore, performance levels [5]. Thus, this period of the day allows for the observation and identification of markers indicating transitions between different conditions. This can provide data for optimizing work and rest schedules in accordance with the tasks and internal regulations of each industry, where employee performance is crucial due to the high cost of human errors [2].

One of the common solutions related to monitoring a person’s condition is the use of technologies that involve the collection and analysis of heart-related data. Portable wearable sensors allow for the recording of electrocardiographic signals and provide insights into a person’s condition [16]. The advantage of methods related to heart activity monitoring in assessing a person’s condition lies in their greater accuracy in detection, as they directly analyze physiological indicators. Moreover, modern solutions based on wearable sensors enable conducting long-term studies in natural activity settings, such as during emergency response training [17], heavy industry work [18], and investigating occupational stress among healthcare personnel during their work shifts [19].

Various heart rate variability (HRV) indices are calculated in studies focusing on sleepiness or drowsiness, including those in the time, frequency, and non-linear domains. The latter ones are employed in applied research to develop fatigue detectors [20] or drowsiness detectors [21]. A significant portion of research is conducted within the context of driving tasks. During simulated driving [22,23] and real driving situations [23], the variance in HRV in the low-frequency (LF) to high-frequency band (HF) ratio (i.e., the sympatho-vagal index) was lower in drowsy conditions compared to awake conditions, as detected by an external observer. In a study involving continuous and monotonous driving, fatigue was accompanied by a decrease in the LH to HF ratio [24]. However, other studies have reported inverse patterns. Resisting sleep to maintain the driving task resulted in an increase in the LH to HF ratio [25]. In a highway driving task characterized by a monotonous environment and a constant speed, a positive correlation was found between the LH to HF ratio and subjective sleepiness (as measured by the KSS) [26]. The inconsistencies in the results of different studies can be attributed to variations in experimental protocols, leading to different intentions and motivations among the subjects. According to a hypothetical classification of subject conditions based on LF and HF power band analysis [23], a high intent to stay awake during sleepiness may result in a ‘stressed’ condition, characterized by high LF band power and low HF band power. Consequently, this leads to high sympatho-vagal index values (LF to HF ratio).

The aforementioned logic aligns with the perspective that sleep deprivation acts as a stressor [27] and that the intention to perform activities requires heightened sympathetic activation [28,29] and reduced parasympathetic activation [29]. Sleep deprivation, whether partial or total, is a commonly employed protocol in the study of sleepiness, drowsiness, and fatigue.

During a partial sleep deprivation period, where participants slept only 3 h per day while carrying out their daily activities, measures of cardiac parasympathetic activity such as square root of the mean of the sum of the squares of differences between adjacent NN intervals (rMSSD) and HF band power decreased, while KSS scores and normalized LF band power increased over a span of three days [30]. In a 40-h study involving total sleep deprivation, KSS scores, standard deviation of the NN intervals (SDNN), variance in HRV in the very low-frequency band (VLF), and LF were positively correlated with vigilance lapses in the Psychomotor Vigilance Test (PVT) [31]. Hence, in a condition of diminished vigilance, LF was higher. This observation suggests that participants exhibited a strong motivation to mobilize themselves, leading to heightened sympathetic activation and a ‘stressed’ condition [23]. This proposition finds support in the results of another study [32], where the authors found a positive correlation between LF to HF ratio and subjective alertness within the first 13 h of sleep deprivation, and a positive correlation with PVT reaction time (indicating a decline in performance) within 14–43 h of sleep deprivation. Regardless, high motivation and the desire to appear alert result in an increased sympatho-vagal index. However, after an extended period of sleep deprivation (14 h or more), the body may lack the necessary resources to sustain optimal performance.

In a separate study involving shift work with an average duration of 13.8 h among wildland firefighters, inverse correlations were identified between HRV (rMSSD) and sleepiness and fatigue [33]. Therefore, heightened subjective sleepiness was associated with reduced parasympathetic activation, indicating that attempting to work under conditions of sleepiness primarily leads to increased sympathetic activation rather than parasympathetic activation (prior to the ‘critical’ 14-h sleep deprivation period).

Shift work involves working outside the typical daytime hours of approximately 8 a.m. to 6 p.m., which disrupts normal circadian rhythms [34]. During work shifts, individuals may strive to maintain alertness artificially, depleting their resources. However, motivation to remain alert during a work shift is always present, even though resources are finite. Hence, it is crucial to examine the body’s functioning outside the explicit motivation to stay alert, specifically in a natural environment when individuals voluntarily construct their evening routines (outside of work shifts). In work shift scenarios, individuals may unknowingly underestimate their subjective sleepiness, as they believe they are alert, potentially skewing the results of sleepiness-related studies. By investigating the body’s performance at different time points in the evening in relation to varying levels of subjective sleepiness, we can obtain more objective data about performance at different sleepiness levels. If the study is conducted at home, without the influence of an experimenter, there is less inclination to distort the assessment of sleepiness.

To conduct such a study, it is needed to choose an experimental timeline. Shift work encompasses any work conducted outside the standard daytime hours of approximately 8 a.m. to 6 p.m. [35], and it is widely acknowledged that shift work disrupts the circadian system [34]. Within the circadian rhythm framework, two distinct stages can be identified: daytime (6 a.m.–8 p.m.) and nighttime (8 p.m.–6 a.m.) [36], separated by the transitional point of the biological evening, occurring around 8 p.m. [37,38,39]. Thus, 8 p.m. can be considered as the time when the body transitions from an active to a passive regime. Considering the definition of shift work mentioned earlier [35], it can be inferred that shift workers frequently engage in their professional activities at 8 p.m. Despite the fact that shift work may result in sleep-related disorders and negative health outcomes [40], shift work does not alter the ‘internal circadian machinery’ [41].

We proposed a hypothesis that at the end of the biological daytime period (around 8 P.M.), the intention to resist sleepiness requires increased sympathetic activation. Conversely, after a biological evening, high sleepiness should be accompanied by parasympathetic activation, as the circadian system allows the body not to resist sleepiness. Building upon Vicente’s et al. (2016) hypothesis regarding four conditions based on the distribution of LF and HF band powers (‘drowsy,’ ‘fatigued,’ ‘awake,’ and ‘stressed’), we argue that high subjective sleepiness during the biological evening should activate the ‘stressed’ condition, while later, high sleepiness should lead to the ‘drowsy’ condition.

The objective of this study was to explore the dynamics of sleepiness-related conditions, starting from the biological evening and continuing at home, without the need for artificial alertness support, i.e., solely performing regular evening household duties.

## 2. Materials and Methods

### 2.1. Study Sample

Recordings for analysis were collected from the Subjective Sleepiness Dynamics Dataset (SSDD [42]). By the time of this paper’s preparation, the total count of participants in the SSDD was equal to 226. One of the inclusion criteria was that people worked on the day of the experiment from 8 a.m. to 6 p.m. or from 9 a.m. to 7 p.m. These were individuals whose professional duties primarily involved computer work and interacting with people outside the home, without significant physical exertion.

Since we wanted to minimize the influence of the factor of going to sleep time, only those participants’ recordings were selected who went to bed between 10:30 p.m. and 11:00 p.m. Also, we selected only those recordings where the time of cyclic tests (the KSS and the SSS) filling out differed no more than 15 min at each time point from the needed time (08:00 p.m., 08:30 p.m., 09:00 p.m., 09:30 p.m., and 10:00 p.m.). And the last criterion was the quality of heart rate data. We selected only those recordings where there was at least a 4 min breakless heart rate recording at each time of cyclic tests. Finally, for the purpose of the current study, 32 recordings from the SSDD were collected for the analysis. All 32 recordings were collected in November–December 2023, and these participants reside in the same region.

As there were 32 selected participants, and each of them had heart rate metrics and KSS/SSS scores for five time points, the entire dataset for analysis consisted of 32 × 5 = 160 observations. During the analysis of the research results, grouping and selection of observations were applied based on time and the level of subjective sleepiness, as indicated in the relevant sections of Section 3.

### 2.2. Apparatus and Web Application

To collect sociodemographic information and data on sleepiness, a web application UnnCyberpsy was developed. UnnCyberpsy was developed using the PHP programming language and was based on the modern microframework ‘CodeIgniter’ version 4 (British Columbia Institute of Technology, British Columbia, Canada). MariaDB RDBMS was selected as the tool for data storage.

For recording the sequence of heart rate intervals, the Polar H10 sensor (Polar Electro Oy, Kempele, Finland), along with the Pro Strap belt (Polar Electro Oy, Kempele, Finland), was utilized. The validity of the Polar H10 sensor has been demonstrated in several studies (e.g., [43,44]). The Polar Sensor Logger App v. 0.25 (Jukka Happonen, Helsinki, Finland), which is based on the Polar SDK, was installed on a Samsung A23 smartphone. This app was used to receive the signal via Bluetooth from the Polar H10 sensor. The collected data was stored on the smartphone and then transferred to a laptop for further analysis.

### 2.3. Study Design

To attract participants for the study, information about the planned research was sent to regional news portals. Interested individuals submitted applications through a Google Form, where they provided their contact information, age, and gender. Subsequently, the timing of their visit to the laboratory for equipment distribution and instructions was coordinated with the participants.

**Figure 1:**
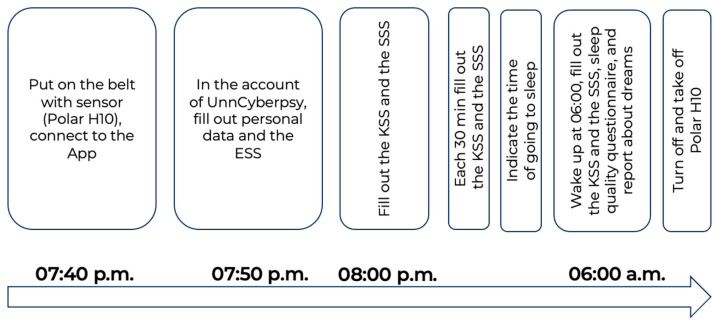
Experimental design.

The full description of the study design is provided in [42] and demonstrated in Figure 1.

The participants connected the Polar H10 sensor to the Polar Sensor Logger App at 07:40 p.m. Then, they filled out personal information and some questionnaires. At 08:00 p.m., and each 30 min further, they filled out cyclic tests (the KSS and the SSS) until the time they went to bed. Participants of the study were instructed that after the start of the experiment, they should remain at home, carry out their regular household duties, and refrain from engaging in any physical exercises.

### 2.4. Data Analysis

Data was preprocessed in the Jupyter Notebook within the Anaconda 2020.07 (Python 3.8.3 64-bit) distribution (Anaconda Inc., Austin, TX, USA). For the purpose of data filtering, NN intervals with duration below 400 msec and above 1300 msec, as well as the ones differing more than 70% of the median of 5 intervals before were removed from the analysis. 4 min heart rate recordings (NN intervals) were selected for each time point for each participant. The ‘hrv-analysis’ module was used to calculate the time- (mean_nni, sdnn, sdsd, nni_50, pnni_50, nni_20, pnni_20, rmssd, median_nni, range_nni, cvsd, cvnni, mean_hr, max_hr, min_hr, std_hr) and frequency-domain metrics (lf, hf, lf_hf_ratio, lfnu, hfnu, total_power, vlf), as well as non-linear-domain ones (csi, cvi, Modified_csi, sampen), at each time point (08:00 p.m., 08:30 p.m., 09:00 p.m., 09:30 p.m., and 10:00 p.m.). The description of metrics is provided in Table A1 (Appendix A).

Statistical analysis was performed using the ‘scipy.stats’ module in the Jupyter Notebook. The independent *t*-test was used to assess differences in heart rate metrics between different levels of subjective sleepiness, as measured by the KSS and the SSS. The Mann-Whitney U test was used to assess differences in heart rate metrics between different levels of subjective sleepiness within different time points. The Pearson criterion was used to calculate the correlations between heart rate metrics and KSS and SSS scores at each time point. The choice of non-parametric criteria was justified by the small size of the subsamples, which were suitable for the selected statistical methods.

## 3. Results

### 3.1. General Data Description

Out of 32 participants, 24 were females. The detailed age–sex distribution of participants is presented in Table 1.

Despite the fact that the sample was not balanced by age and sex, this did not contradict the purpose of the study as we wanted to examine some general effects of subjective sleepiness on heart rate data.

**Figure 2:**
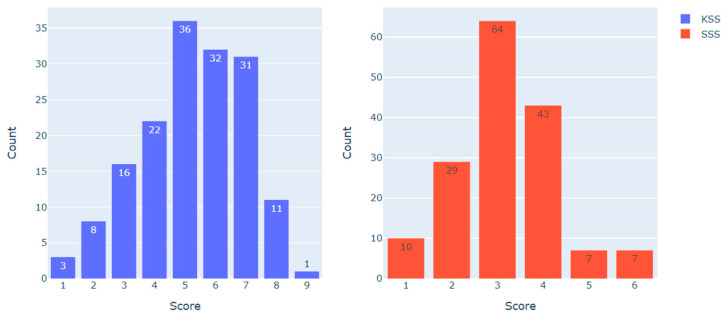
KSS and SSS scores distribution in the dataset.

Figure 2 demonstrates the distribution of KSS and SSS scores in the dataset (*N* = 160).

To examine the heart rate metrics associated with different scores on the KSS and the SSS, the following data sampling approach was employed. For the KSS, observations with less than 5 points (indicating ‘low’ sleepiness, *N* = 49) and more than 6 points (indicating ‘high’ sleepiness, *N* = 43) were compared. For the SSS, observations with less than 3 points (indicating ‘low’ sleepiness, *N* = 39) and more than 3 points (indicating ‘high’ sleepiness, *N* = 57) were compared. Observations with scores of 5–6 on the KSS (*N* = 68) and 3 on the SSS (*N* = 64) were excluded from the analysis, as they represented the category of ‘medium sleepiness’. The resulting distribution is presented in Table 2.

### 3.2. Heart Rate Metrics Comparison in Different Levels of Subjective Sleepiness

First, we analyzed the difference in HRV metrics between ‘low’ and ‘high’ sleepiness, independent of time. These data were subsequently compared with the time-associated data.

Table 3 and Table 4 represent significant differences and trends found for heart rate metrics for the KSS and SSS, respectively.

The data presented in Table 3 indicate that there was a significant difference in nni_20 between ‘high’ and ‘low’ sleepiness (t = 2.37; *p* = 0.020), as assessed by the KSS. Additionally, certain trends were observed: nni_50 (t = 1.68; *p* = 0.097) and pnni_20 (t = 1.71; *p* = 0.091) showed lower values, while lf_hf_ratio (t = 1.69; *p* = 0.094) exhibited higher values in ‘high’ sleepiness compared to ‘low’ sleepiness.

The data presented in Table 4 indicate that the lf_hf_ratio was found to be higher in ‘high’ sleepiness compared to ‘low’ sleepiness (t = 2.03; *p* = 0.045), as measured by the SSS. Additionally, a trend towards lower nni_20 was observed for ‘high’ sleepiness compared to ‘low’ sleepiness (t = 1.89; *p* = 0.062).

### 3.3. Heart Rate Metrics Comparison in Different Levels of Subjective Sleepiness within Different Time Points

To further specify the characteristics of HRV metrics during ‘low’ and ‘high’ sleepiness, we conducted a comparative analysis taking into account the factor of time (at each time point: 08:00 p.m., 08:30 p.m., 09:00 p.m., 09:30 p.m., and 10:00 p.m.).

Significant differences and trends in heart rate metrics between ‘low’ and ‘high’ sleepiness were observed specifically at the 08:30 p.m. and 09:00 p.m. time points. Table 5 and Table 6 present the significant differences and trends identified for heart rate metrics at these specific time points for the KSS and SSS, respectively.

The data presented in Table 5 indicate that at the 08:30 p.m. time point, heart rate variability metrics were lower in ‘high’ sleepiness, as measured by the KSS. Specifically, the minimum heart rate (min_hr) was higher in ‘high’ sleepiness (U = 12; *p* = 0.049). As for frequency domain metrics, lf (U = 5; *p* = 0.004), total_power (U = 2; *p* = 0.001), and vlf (U = 2; *p* = 0.001) appeared to be significantly lower in ‘high’ sleepiness. Additionally, the following trends were observed: lf_hf_ratio and lfnu were lower (U = 15; *p* = 0.095 for both), while hfnu was higher (U = 50; *p* = 0.095) in ‘high’ sleepiness. Significant differences were also found for non-linear domain metrics, with modified_csi and cvi being lower (U = 6; *p* = 0.007 for modified_csi, U = 9; *p* = 0.019 for cvi), and samplen being higher (U = 5; *p* = 0.004) in ‘high’ sleepiness.

Regarding the SSS, significant differences or trends were observed at two time points (08:00 p.m. and 08:30 p.m.), as shown in Table 6. At 08:00 p.m., only frequency domain metrics were found to be sensitive to the level of sleepiness: lf_hf_ratio (U = 28; *p* = 0.091) and lfnu (U = 28; *p* = 0.091) were higher, while hfnu (U = 28; *p* = 0.091) was lower in ‘high’ sleepiness. At 08:30 p.m., various domain metrics were identified as sensitive to the level of sleepiness. Total_power (U = 15; *p* = 0.027) and cvi (U = 4.49; *p* = 4.2) were lower in ‘high’ sleepiness. There was also a trend towards lower vlf values in ‘high’ sleepiness.

### 3.4. Heart Rate Metrics Correlations with Sleepiness Scores within Different Time Points

To conduct a more detailed analysis of the relationship between HRV metrics and subjective sleepiness, we performed a correlation analysis between them. No significant correlations were found for the whole dataset.

For a more in-depth exploration of the relationship between HRV metrics and subjective sleepiness, we examined their correlations at each of the time points. The results of correlation analysis within different time points are presented in Table 7.

Thus, significant correlations were found only at 08:30 p.m. and at 09:00 p.m. At 08:30 p.m., range_nni was negatively correlated with KSS score (R = −0.392; *p* = 0.027) and with SSS score (R = −0.382; *p* = 0.031). KSS score was negatively correlated with lf (R = −0.350; *p* = 0.049), total_power (R = −0.357; *p* = 0.045), and vlf (R = −0.385; *p* = 0.029). At 09:00 p.m., hf was positively correlated with KSS score (R = 0.394; *p* = 0.026).

## 4. Discussion

Our data analysis revealed that during the biological evening (8 p.m.), only frequency domain metrics showed a tendency to differ between levels of subjective sleepiness. Specifically, lf and lfnu tended to be higher, while hfnu tended to be lower in ‘high’ sleepiness compared to ‘low’ sleepiness. In the introduction, we hypothesized that high subjective sleepiness during the biological evening would activate a ‘stressed’ condition because the body perceives this time as being awake. Resisting sleepiness at 8 p.m. would require self-control. The fact that only frequency domain metrics were correlated with sleepiness can be explained by previous findings that fMRI activity in brain regions, including the dorsolateral prefrontal cortex, is correlated with cardiovagal activity (HF) [45], and that stimulation of the dorsolateral prefrontal cortex affects muscle sympathetic nerve activity [46]. Moreover, the dorsolateral prefrontal cortex is involved in self-control [47]. Thus, our results indirectly support the idea that resisting sleepiness at 8 p.m. requires self-control. In ‘high’ sleepiness at 8 p.m., sympathetic activation (lhnu) increased, parasympathetic activation (fhnu) decreased, and the sympatho-vagal index (lf_hf_ratio) increased, indicating a ‘stressed’ condition [23].

Starting at 8:30 p.m. (after the biological evening), our analysis revealed that ‘high’ sleepiness was associated with a ‘drowsy’ condition [23]. Sympathetic activation (lf and lfnu) decreased, as did parasympathetic activation (hf and hfnu). Additionally, our results showed that at 8:30 p.m., ‘high’ subjective sleepiness was accompanied by lower time-domain metrics, indicating a decrease in heart rate variability. This finding is consistent with previous research linking reduced variability metrics to sleepiness, fatigue [33], and lack of vigilance [31]. Moreover, non-linear domain metrics (cvi, Modified_csi, and samplen) demonstrated sensitivity to the level of subjective sleepiness at 8:30 p.m., which is noteworthy as these metrics are seldom calculated in fundamental sleepiness research. For example, cvi has been observed to decrease during inhalant anesthesia and increase upon regaining consciousness [48], suggesting its association with metabolic demands [49]. Therefore, in our study, a decrease in cvi in ‘high’ sleepiness after the biological evening might indicate the unconscious body’s effort to perform vital functions. Similarly, Modified_csi, commonly used in detecting epileptic seizures [50,51], is considered an indicator of sympathetic ‘overdrive’ [52]. In our study, Modified_csi was lower in ‘high’ sleepiness after the biological evening, aligning with the decrease in lf. Furthermore, samplen, associated with poor performance during PVT [53], was higher in ‘high’ sleepiness after the biological evening, indicating reduced vigilance.

At 9:00 p.m., only the correlation analysis yielded significant results. Parasympathetic activation (hf) exhibited a positive correlation with the SSS score. This suggests that sympathetic activation had already decreased, and the increase in the ‘drowsy’ condition at 9:00 p.m. was primarily driven by an elevation in vagal activation. This finding can be utilized to further refine approaches that differentiate types of ‘drowsy’ conditions based on the level of parasympathetic activation, building upon Vincent et al.’s prior work [23].

After 9:00 p.m. until 10:00 p.m., no significant results were revealed, neither in correlation nor in differences analysis. Perhaps after 9:00 p.m., the participants transitioned to a nighttime regimen, and they were all sufficiently sleepy and prepared for falling asleep. In our previous study [42], we demonstrated that starting at 9:00 p.m., there was a significant increase in subjective sleepiness across all the SSDD. Other studies have reported similar sleepiness dynamics. A study of sleepiness dynamics in helicopter pilots showed a steady increase in subjective sleepiness after 8:00 p.m. (KSS) [13]. The level of subjective sleepiness in fishermen of different vessel types increased towards the evening [3]. The level of subjective sleepiness in fishermen of different types of vessels increased in the evening [54]. Thus, our suggestions and results align with the findings of other studies.

Within the current study, high sleepiness at 08:00 p.m. was considered as a ‘stressed’ condition and ‘high’ sleepiness at 08:30 p.m. and at 09:00 p.m. as a ‘drowsy’ condition. In Table 8, we accumulate the results of the current study by distinguishing heart rate metrics associated with ‘high’ sleepiness in ‘stressed’ and ‘drowsy’ conditions. These results should be taken into account while researching sleepiness, fatigue, or vigilance in shift workers.

Additionally, it is necessary to discuss the results of comparing heart rate metrics without considering the specific time points. The findings indicated a decrease in time-domain variability metrics and an increase in the sympatho-vagal index (lf_hf_ratio) in ‘high’ sleepiness. The observed dynamics of time-domain variability metrics align with those reported in other studies [31,33]. However, regarding the sympatho-vagal index, our results contradict the findings in [22,24] but support the results reported in [25,26]. This suggests that subjective sleepiness alone cannot fully describe the condition, and other domains, such as the circadian clock, should be considered in research on shift work conditions. Such a conclusion is supported by the evidence that drowsiness-related brain responses were affected by the time of the day [55]. For accurate and objective condition detection in shift workers, it is crucial to take into account circadian rhythms.

The obtained results could be utilized for the development of automated solutions to monitor the condition of shift workers. For the creation of precise algorithms, it’s important to take into account the biological time and the fact that high subjective sleepiness can be associated with different conditions—both ‘stressed’ and ‘drowsy’. We demonstrated that at 8 p.m., individuals are more resistant to sleepiness, and in such cases, high sleepiness is accompanied by a ‘stressed’ condition. Later, high sleepiness leads to a ‘drowsy’ condition, which involves different physiological correlates.

## 5. Limitations

The primary limitation of this study is linked to the small sample sizes used for comparative and correlational analysis within different time points. Nevertheless, we made an effort to address this limitation by employing non-parametric statistics. This study was exploratory, and in the future, we plan to incorporate a larger portion of data from the dataset to facilitate a more complex analysis.

Additionally, we intend to apply machine learning methods to develop a heartbeat-based drowsiness detector. We have not yet taken into account the types of professional activities of the selected participants in the study. However, we focused on those whose professional activities were similar in terms of activity levels.

Within our study, we divided our sample into sleepiness levels without relying on any pre-established classification. However, the decision to divide sleepiness based on KSS and SSS scores was made considering the distribution of the data. Our goal was to ensure a relatively equal count of ‘low’ and ‘high’ sleepiness data that would be suitable for analysis.

Another limitation is that we did not account for within-subject effects and instead conducted independent tests during the comparison. However, our intention was to capture general effects without considering individual variations.

The aim of the current study was to investigate sleepiness-related conditions in people in general, but we are directing our findings towards shift workers. Of course, having a separate sample of shift workers would be valuable, and we intend to do so in the near future. However, considering that shift work itself does not influence the ‘internal circadian machinery,’ we believe that our findings can be taken into account while studying sleepiness-related conditions in shift workers.

## 6. Conclusions

Subjective sleepiness may be accompanied by different conditions. At the time of biological evening, high sleepiness was associated with a ‘stressed’ condition with higher sympathetic activation, while later, it was associated with a ‘drowsy’ condition with higher parasympathetic activation and a decline in variability in heart rate. Our results provide evidence that condition type in high sleepiness depends on biological time. This should be taken into account in managing work regimes in shift work and while creating alertness detectors.

## Figures and Tables

**Table 1:** Age and sex distribution of the dataset.

Age	Sex	*N*
<25	male	9
	female	2
<35	male	9
	female	2
35 and more	male	6
	female	4

**Table 2:** The distribution of selected observations according to time points and sleepiness levels.

Time Point	KSS		SSS	
	‘Low’ Sleepiness	‘High’ Sleepiness	‘Low’ Sleepiness	‘High’ Sleepiness
20:00:00	13	4	15	7
20:30:00	13	5	10	8
21:00:00	9	7	6	9
21:30:00	8	13	5	16
22:00:00	6	14	3	17
Total	49	43	39	57

**Table 3:** Heart rate metrics mean values in different levels of subjective sleepiness, as measured by the KSS (t—the value of independent *t*-test, *p*—*p*-value).

Domain	Metric	KSS Score < 5 (*N* = 49)	KSS Score > 5 (*N* = 43)	t	*p*
Time	nni_50	46.41	33.26	1.68	0.097
	nni_20	140.71	115.47	2.37	0.020
	pnni_20	47.94	40.6	1.71	0.091
Frequency	lf_hf_ratio	3.8	4.9	1.69	0.094

**Table 4:** Heart rate metrics mean values in different levels of subjective sleepiness, as measured by the SSS (t—the value of independent *t*-test, *p*—*p*-value).

Domain	Metric	SSS Score < 3 (*N* = 39)	SSS Score > 3 (*N* = 57)	t	*p*
Time	nni_20	134.26	114.33	1.89	0.062
Frequency	lf_hf_ratio	3.75	5.47	2.03	0.045

**Table 5:** Heart rate metrics mean values in different levels of subjective sleepiness within time point 08:30 p.m., as measured by the KSS (U—the value of Mann-Whitney criterion, *p*—*p*-value).

Domain	Metric	‘Low’ Sleepiness (N = 13)	‘High’ Sleepiness (N = 5)	U	*p*
Time	sdnn	68.86	38.21	7	0.010
	sdsd	37.2	24.27	11	0.035
	rmssd	37.2	24.27	11	0.035
	range_nni	358.92	205.2	4.5	0.007
	cvsd	0.05	0.03	13	0.059
	cvnni	0.09	0.05	6	0.007
	min_hr	63.32	71.22	12	0.049
	std_hr	7.21	4.21	8	0.014
Frequency	lf	1364.96	487.1	5	0.004
	lf_hf_ratio	4.27	2.82	15	0.095
	lfnu	78.21	70.5	15	0.095
	hfnu	21.79	29.5	15	0.095
	total_power	3106.39	968	2	0.001
	vlf	1303.23	291.76	2	0.001
Non-Linear	cvi	4.55	4.12	9	0.019
	Modified_csi	1446.96	619.03	6	0.007
	sampen	1.21	1.56	5	0.004

**Table 6:** Heart rate metrics in different levels of subjective sleepiness within time points 08:00 p.m. and 08:30 p.m., as measured by the SSS (U—the value of Mann-Whitney criterion, *p*—*p*-value).

Domain	Metric	‘Low’ Sleepiness (*N* = 15)	‘High’ Sleepiness (*N* = 7)	U	*p*
08:00 p.m.					
Frequency	lf_hf_ratio	4.13	9.31	28	0.091
	lfnu	77.82	84.44	28	0.091
	hfnu	22.18	15.56	28	0.091
**Domain**	**Metric**	**‘Low’** **Sleepiness (*N* = 10)**	**‘High’ Sleepiness** **(*N* = 8)**	**U**	***p***
08:30 p.m.					
Time	sdsd	36.97	24.88	14	0.021
	nni_50	39.8	17.38	18.5	0.062
	pnni_50	15.48	5.7	20	0.083
	pnni_20	47.97	32.86	19	0.068
	rmssd	36.97	24.88	14	0.021
	range_nni	321.7	236.25	15.5	0.033
	cvnni	0.07	0.06	20	0.083
Frequency	total_power	2545.7	1390.07	15	0.027
	vlf	841	526.46	18	0.055
Non-Linear	cvi	4.49	4.2	17	0.043

**Table 7:** Heart rate metrics significant correlations with KSS and SSS scores within different time points (*p*—*p*-value).

Time point	Domain	Metric	KSS Score		SSS Score	
			Pearson R	*p*	Pearson R	*p*
08:30 p.m.	Time	range_nni	−0.392	0.027		
	Frequency	lf	−0.350	0.049	−0.382	0.031
		total_power	−0.357	0.045		
		vlf	−0.385	0.029		
09:00 p.m.	Frequency	hf			0.394	0.026

**Table 8:** Heart rate metrics dynamics related to ‘high’ subjective sleepiness in ‘stressed’ and ‘drowsy’ conditions.

Domain	Metrics	Condition	
		Stressed	Drowsy
Frequency	lf/lhnu	increase	decrease
	hf/hfnu	decrease	increase
	lf_hf_ratio	increase	decrease
Time	variability	-	decrease
Non-Linear	cvi	-	decrease
	Modified_csi	-	decrease
	sampen	-	increase

## Data Availability

The data presented in this study are available on request from the corresponding author. The data are not publicly available due to their containing information that could compromise the privacy of research participants.

## Appendix A

**Table A1:** The description of heart rate variability metrics (partially taken from [21]).

Domain	Metrics	Description
Time	mean_nni	mean of the NN intervals
	sdnn	standard deviation of the NN intervals
	sdsd	standard deviation of differences between adjacent NN intervals
	nni_50	intervals’ number differences of successive NN intervals greater than 50 ms
	pnni_50	derived proportion by dividing nni_50 by the NN intervals’ total number.
	nni_20	intervals’ number differences of successive NN intervals greater than 20 ms
	pnni_20	derived proportion by dividing nni_20 by the r–r intervals’ total number
	rmssd	square root of the mean of the sum of the squares of differences between adjacent NN intervals
	median_nni	median of the NN intervals
	range_nni	difference between the maximum and the minimum of the NN intervals
	cvsd	rmssd divided mean_nni
	cvnni	sdnn divided by mean_nni
	mean_hr	heart rate mean
	max_hr	heart rate maximum
	min_hr	heart rate minimum
	std_hr	standard deviation of the heart rate
Frequency	lf	variance in hrv in the low-frequency band (0.03–0.15 Hz)
	hf	variance in hrv in the high-frequency band (0.18–0.45 Hz)
	lf_hf_ratio	lf to hf ratio, sympatho-vagal index
	lfnu	derived proportion by dividing lf by the sum of lf and hf
	hfnu	derived proportion by dividing hf by the sum of lf and hf
	total_power	total power density spectral
	vlf	variance in hrv in the very low-frequency band (0.00–0.03 Hz)
Non-Linear	csi	cardiac sympathetic index
	cvi	cadiac vagal index
	modified_csi	modified csi
	sampen	sample entropy

## References

[1] Baulk S.D., Fletcher A., Kandelaars K.J., Dawson D., Roach G.D. (2009). A field study of sleep and fatigue in a regular rotating 12-h shift system. Appl. Ergon..

[2] Ganesan S., Magee M., Stone J.E., Mulhall M.D., Collins A., Howard M.E., Sletten T.L. (2019). The impact of shift work on sleep, alertness and performance in healthcare workers. Sci. Rep..

[3] Abrahamsen A., Weihe P., Debes F., van Leeuwen W.M. (2022). Sleep, sleepiness, and fatigue on board Faroese fishing vessels. Nat. Sci. Sleep.

[4] Åkerstedt T., Axelsson J., Lekander M., Orsini N., Kecklund G. (2012). The daily variation in sleepiness and its relation to the preceding sleep episode-a prospective study across 42 days of normal living. J. Sleep Res..

[5] Shochat T., Santhi N., Herer P., Dijk D., Skeldon A.C. (2021). Sleepiness is a signal to go to bed: Data and model simulations. Sleep.

[6] Bendaoud I., Etindele Sosso F.A. (2022). Socioeconomic Position and Excessive Daytime Sleepiness: A Systematic Review of Social Epidemiological Studies. Clocks Sleep.

[7] Yu Y.-K., Yao Z.-Y., Wei Y.-X., Kou C.-G., Yao B., Sun W.-J., Li S.-Y., Fung K., Jia C.-X. (2022). Depressive Symptoms as a Mediator between Excessive Daytime Sleepiness and Suicidal Ideation among Chinese College Students. Int. J. Environ. Res. Public Health.

[8] Carskadon M.A., Dement W.C. (1977). Sleepiness and Sleep State on a 90-Min Schedule. Psychophysiology.

[9] Åkerstedt T., Gillberg M. (1990). Subjective and objective sleepiness in the active individual. Int. J. Neurosci..

[10] Cai A.W., Manousakis J.E., Singh B., Kuo J., Jeppe K.J., Francis-Pester E., Anderson C. (2021). On-road driving impairment following sleep deprivation differs according to age. Sci. Rep..

[11] Santhi N., Horowitz T.S., Duffy J.F., Czeisler C.A. (2007). Acute Sleep Deprivation and Circadian Misalignment Associated with Transition onto the First Night of Work Impairs Visual Selective Attention. PLoS ONE.

[12] Muck R.A., Hudson A.N., Honn K.A., Gaddameedhi S., Van Dongen H.P.A. (2022). Working around the Clock: Is a Person’s Endogenous Circadian Timing for Optimal Neurobehavioral Functioning Inherently Task-Dependent?. Clocks Sleep.

[13] Flaa T.A., Bjorvatn B., Pallesen S., Zakariassen E., Harris A., Gatterbauer-Trischler P., Waage S. (2022). Sleep and sleepiness measured by diaries and actigraphy among Norwegian and Austrian helicopter emergency medical service (HEMS) pilots. Int. J. Environ. Res. Public Health.

[14] Sunde E., Mrdalj J., Pedersen T., Thun E., Bjorvatn B., Grønli J., Pallesen S. (2020). Role of nocturnal light intensity on adaptation to three consecutive night shifts: A counterbalanced crossover study. Occup. Environ. Med..

[15] Manousakis J.E., Mann N., Jeppe K.J., Anderson C. (2021). Awareness of sleepiness: Temporal dynamics of subjective and objective sleepiness. Psychophysiology.

[16] Rogers B., Schaffarczyk M., Gronwald T. (2022). Estimation of Respiratory Frequency in Women and Men by Kubios HRV Software Using the Polar H10 or Movesense Medical ECG Sensor during an Exercise Ramp. Sensors.

[17] Koizumi N., Ogata H., Negishi Y., Nagayama H., Kaneko M., Kiyono K., Omi N. (2023). Energy Expenditure of Disaster Relief Operations Estimated Using a Tri-Axial Accelerometer and a Wearable Heart Rate Monitor. Int. J. Environ. Res. Public Health.

[18] Delling A.C., Jakobsmeyer R., Coenen J., Christiansen N., Reinsberger C. (2023). Home-Based Measurements of Nocturnal Cardiac Parasympathetic Activity in Athletes during Return to Sport after Sport-Related Concussion. Sensors.

[19] Hamidi Shishavan H., Garza J., Henning R., Cherniack M., Hirabayashi L., Scott E., Kim I. (2023). Continuous physiological signal measurement over 24-hour periods to assess the impact of work-related stress and workplace violence. Appl. Ergon..

[20] Nasirzadeh F., Mir M., Hussain S., Tayarani Darbandy M., Khosravi A., Nahavandi S., Aisbett B. (2020). Physical Fatigue Detection Using Entropy Analysis of Heart Rate Signals. Sustainability.

[21] Antunes A.R., Braga A.C., Gonçalves J. (2023). Drowsiness Transitions Detection Using a Wearable Device. Appl. Sci..

[22] Awais M., Badruddin N., Drieberg M. (2017). A hybrid approach to detect driver drowsiness utilizing physiological signals to improve system performance and wearability. Sensors.

[23] Vicente J., Laguna P., Bartra A., Bailon R. (2016). Drowsiness detection using heart rate variability. Med. Biol. Eng. Comput..

[24] Patel M., Lal S.K.L., Kavanagh D., Rossiter P. (2011). Applying neural network analysis on heart rate variability data to assess driver fatigue. Expert Syst. Appl..

[25] Furman G.D., Baharav A., Cahan C., Akselrod S. Early detection of falling asleep at the wheel: A Heart Rate Variability approach. Proceedings of the IEEE Computers in Cardiology.

[26] Zhang N., Fard M., Bhuiyan M.H.U., Verhagen D., Azari M.F., Robinson S.R. (2018). The effects of physical vibration on heart rate variability as a measure of drowsiness. Ergonomics.

[27] McEwen B.S., Karatsoreos I.N. (2015). Sleep Deprivation and Circadian Disruption: Stress. Allostasis, and Allostatic Load. Sleep Med. Clin..

[28] Zhong X., Hilton H.J., Gates G.J., Jelic S., Stern Y., Bartels M.N. (1985). Increased sympathetic and decreased parasympathetic cardiovascular modulation in normal humans with acute sleep deprivation. J. Appl. Physiol..

[29] Morales J., Yáñez A., Fernández-González L., Montesinos-Magraner L., Marco-Ahulló A., Solana-Tramunt M. (2019). Stress and autonomic response to sleep deprivation in medical residents: A comparative cross-sectional study. PLoS ONE.

[30] Bourdillon N., Jeanneret F., Nilchian M., Albertoni P., Ha P., Millet G.P. (2021). Sleep Deprivation Deteriorates Heart Rate Variability and Photoplethysmography. Front. Neurosci..

[31] Chua E.C.P. (2012). Heart rate variability can be used to estimate sleepiness-related decrements in psychomotor vigilance during total sleep deprivation. Sleep.

[32] Citi L. Point process heart rate variability assessment during sleep deprivation. Proceedings of the IEEE Computing in Cardiology.

[33] Jeklin A.T., Perrotta A.S., Davies H.W., Bredin S.S.D., Paul D.A., Warburton D.E.R. (2021). The association between heart rate variability, reaction time, and indicators of workplace fatigue in wildland firefighters. Int. Arch. Occup. Environ. Health.

[34] Boivin D.B., Boudreau P., Kosmadopoulos A. (2021). Disturbance of the Circadian System in Shift Work and Its Health Impact. J. Biol. Rhythm..

[35] Rivera A.S., Akanbi M., O’Dwyer L.C., McHugh M. (2020). Shift work and long work hours and their association with chronic health conditions: A systematic review of systematic reviews with meta-analyses. PLoS ONE.

[36] Reid K.J., Santostasi G., Baron K.G., Wilson J., Kang J., Zee P.C. (2014). Timing and intensity of light correlate with body weight in adults. PLoS ONE.

[37] Scheer F.A., Morris C.J., Shea S.A. (2013). The internal circadian clock increases hunger and appetite in the evening independent of food intake and other behaviors. Obesity.

[38] Morris C.J., Garcia J.I., Myers S., Yang J.N., Trienekens N., Scheer F.A. (2015). The Human Circadian System Has a Dominating Role in Causing the Morning/Evening Difference in Diet-Induced Thermogenesis. Obesity.

[39] Noh J. (2018). The Effect of Circadian and Sleep Disruptions on Obesity Risk. J. Obes. Metab. Syndr..

[40] Jehan S., Zizi F., Pandi-Perumal S.R., Myers A.K., Auguste E., Jean-Louis G., McFarlane S.I. (2017). Shift Work and Sleep: Medical Implications and Management. Sleep Med. Disord. Int. J..

[41] Folkard S. (2008). Do permanent night workers show circadian adjustment? A review based on the endogenous melatonin rhythm. Chronobiol. Int..

[42] Demareva V., Viakhireva V., Zayceva I., Isakova I., Okhrimchuk Y., Zueva K., Demarev A., Nazarov N., Edeleva J. (2023). Temporal dynamics of subjective sleepiness: A convergence analysis of two scales. Biol. Rhythm Res..

[43] Schaffarczyk M., Rogers B., Reer R., Gronwald T. (2022). Validity of the Polar H10 Sensor for Heart Rate Variability Analysis during Resting State and Incremental Exercise in Recreational Men and Women. Sensors.

[44] Skála T., Vícha M., Rada M., Vácha J., Flašík J., Táborský M. (2022). Feasibility of evaluation of Polar H10 chest-belt ECG in patients with a broad range of heart conditions. Cor Vasa.

[45] Napadow V., Dhond R., Conti G., Makris N., Brown E.N., Barbieri R. (2008). Brain correlates of autonomic modulation: Combining heart rate variability with fMRI. NeuroImage.

[46] Sesa-Ashton G., Wong R., McCarthy B., Datta S., Henderson L.A., Dawood T., Macefield V.G. (2022). Stimulation of the dorsolateral prefrontal cortex modulates muscle sympathetic nerve activity and blood pressure in humans. Cereb. Cortex Commun..

[47] Kirk U., Harvey A., Montague P.R. (2011). Domain expertise insulates against judgment bias by monetary favors through a modulation of ventromedial prefrontal cortex. Proc. Natl. Acad. Sci. USA.

[48] Donchin Y., Feld J., Porges S. (1985). Respiratory Sinus Arrhythmia during Recovery from Isoflurane—Nitrous Oxide Anesthesia. Anesth. Analg..

[49] Porges S.W. (1995). Cardiac vagal tone: A physiological index of stress. Neurosci. Biobehav. Rev..

[50] Jeppesen J., Beniczky S., Johansen P., Sidenius P., Fuglsang-Frederiksen A. Using Lorenz plot and Cardiac Sympathetic Index of heart rate variability for detecting seizures for patients with epilepsy. Proceedings of the Annual International Conference of the IEEE Engineering in Medicine and Biology Society.

[51] Jeppesen J., Beniczky S., Johansen P., Sidenius P., Fuglsang-Frederiksen A. (2015). Detection of epileptic seizures with a modified heart rate variability algorithm based on Lorenz plot. Seizure.

[52] Jeppesen J., Fuglsang-Frederiksen A., Johansen P. (2019). Seizure detection based on heart rate variability using a wearable electrocardiography device. Epilepsia.

[53] Lin C.T., Nascimben M., King J.T., Wang Y.K. (2018). Task-related EEG and HRV entropy factors under different real-world fatigue scenarios. Neurocomputing.

[54] Hoddes E., Zarcone V., Smythe H., Phillips R., Dement W.C. (1973). Quantification of Sleepiness: A New Approach. Psychophysiology.

[55] Brown T., Johnson R., Milavetz G. (2013). Identifying periods of drowsy driving using EEG. Ann. Adv. Automot. Med..

